# Supplementary material for: Inka Unku: Imperial or provincial? State-local relations
Source: PLoS One. 2023 Feb 8;18(2):e0280511. doi: 10.1371/journal.pone.0280511 (PMC9907846; doi:10.1371/journal.pone.0280511)
Supplement: S1 Table — Register of unku located in museums in Europe, the United States of America and South America. (DOCX) [file pone.0280511.s001.docx]

S1 Table. List of *unku* from around the world.

| **Provenance/Collection** | **Id/Inventory** | **Type/Style^a^** | **Measures** | **Origin** |
| --- | --- | --- | --- | --- |
| American Museum of Natural History, New York | 41.2/7037 | BW (miniature) | 7.8 × 7 cm | Unknown |
| American Museum of Natural History, New York | 41.2/885 | DW | 93.5 x 79 cm | Coast of Peru |
| American Museum of Natural History, New York | 41.2/762 | ZZW | 80.5 x 73 cm | Pinilla, Ocucaje, Ica valley |
| American Museum of Natural History, New York | 41.2/964 | IK | 86 x 72 cm | Peru |
| Boston Museum of Fine Arts | 471.097 | BW | 84.5 x 78 cm | Unknown |
| Brooklyn Museum | 61.2 | IK | 84 x 74 cm | Unknown |
| Brooklyn Museum | 86.224.133 | IK | 84 x 74 cm | Unknown |
| Brooklyn Museum | 411.275.106 | ZZW | 89 x 79 cm | Unknown |
| California Academy of Sciences | 389-2357 | BW | 82.5 x 73.5 cm | Unknown |
| Dallas Museum of Art | 1995.32.McD | BW | 88.3 × 80 cm | Unknown |
| Dumbarton Oaks, Washington DC | PC.B.518 | AOT | 90 × 77 cm | Unknown |
| Etnografiska Museet, Göteborgs | 21.6.9 | IK | No data | Ica? |
| Etnografiska Museet, Stockholm | 1929.26.0149 | IK | 79 x 97 cm | Ica valley |
| Ethnologishes Museum, Staatliche Museen zu Berlin | VA 16621 | BW | 88 x 76.2 cm | Nazca |
| Ethnologishes Museum, Staatliche Museen zu Berlin | VA 4576 | Provincial Inka/DW and ZZ neck yoke | 65 x 62 cm | Pica, Chile |
| Ethnologishes Museum, Staatliche Museen zu Berlin | VA 21367 | DW | 85.5 x 75.5 cm | Unknown |
| Ethnologishes Museum, Staatliche Museen zu Berlin | VA 16630 | Indefinite | 87.5 x 75 cm | Unknown |
| Ethnologishes Museum, Staatliche Museen zu Berlin | VA 16296 | DW | 83.5 x 79.5 cm | Unknown |
| Ethnologishes Museum, Staatliche Museen zu Berlin | VA 16289 | Provincial Inka/DW and ZZW | 82 x 78 cm | Unknown |
| Ethnologishes Museum, Staatliche Museen zu Berlin | VA Nls 1385 | Provincial Inka?/AOT | 68 x 71 cm | Central Coast |
| Ethnologishes Museum, Staatliche Museen zu Berlin | VA 62696 | Provincial Inka/DW and Diamond neck yoke | 148 x 76 cm | Pachacamac |
| Ethnologishes Museum, Staatliche Museen zu Berlin | VA 66009 | ZZW | 90 x 74 cm | Ica |
| Ethnologishes Museum, Staatliche Museen zu Berlin | VA 29116 | ZZW | 89 x 76 cm | Ica |
| Ethnologishes Museum, Staatliche Museen zu Berlin | VA 51806 | DW (child size) | 49 x 42 cm | Ica |
| Ethnologishes Museum, Staatliche Museen zu Berlin | VA 29107 | DW | 79 x 76 cm | Ica |
| Ethnologishes Museum, Staatliche Museen zu Berlin | VA 31526 | IK | 88 x 70 cm | Chimbote |
| Ethnologishes Museum, Staatliche Museen zu Berlin | VA 29080 | BW | 89 x 76 cm | Ica |
| Ethnologishes Museum, Staatliche Museen zu Berlin | VA 55462 | BW (miniature) | 12.8 x 10 cm | Pachacamac |
| Field Museum of Natural History, Chicago | 171377 | IK (miniature) | 38 x 31 cm | Poroma, cuenca de Nazca, Peru |
| Field Museum of Natural History, Chicago | 1534 | BW | No data | Lima, Ancon |
| Musée de l'Homme, Paris | Berthon Collection | BW | No data | Near Lima |
| Musée du Quai Branly, Paris | 71.1911.21.448 | BW | 90.5 x 78 cm | Near Lima |
| Museo Histórico Provincial de Rosario Dr. Julio Marc | No data | TW | 84 x 74 cm | Ica valley |
| Museo Nacional de Arqueología, Antropología e Historia del Perú, Lima | 7020 | IK | 92 x 76 cm | Armatambo, Lima valley |
| Museo Nacional de Arqueología, Antropología e Historia del Perú, Lima | RT-2377 | BW | 110 x 98 cm | Peru |
| Museo Regional de Ica | No data | TW | 80 x 70 cm | Quebrada de la Vaca |
| Museo Regional de Ica | 713 | BW | 89 x 78 cm | Chavina Acari valley |
| Museo Nacional de Arte, La Paz | No data | DW | No data | Unknown |
| Museo Nacional de Arte, La Paz | No data | BW | No data | Isla del Sol Titicaca, Bolivia |
| Museum Fünf Kontinente, München | X.466 | BW | 95 x 78 cm | Los Majuelos Río Grande de Nazca |
| Museum Fünf Kontinente, München | X.447 | IK | 92 x 76 cm | Río Grande de Nazca |
| Museum Fünf Kontinente, München | No data | IK (miniature) | No data | Ica |
| The Cleveland Museum of Art | 1.957.136 | IK | 86.5 x 76.8 cm | Ica valley |
| The George Washington University Museum and The Textile Museum | TM.1966.7.172 | BW | 89 x 74 cm | Río Grande de Nazca |
| The George Washington University Museum and The Textile Museum | 1966.7.171 | BW | 86 x 74 cm | Rio Grande de Nazca |
| The George Washington University Museum and The Textile Museum | TM.1966.59.28 | IK | 84 x 71 cm | Unknown |
| The George Washington University Museum and The Textile Museum | 91.147 | IK | 95 x 78.5 cm | Ica valley |
| The George Washington University Museum and The Textile Museum | 1960.13.7 | TW | No data x 79 cm | Unknown |
| The George Washington University Museum and The Textile Museum | 1964.12.2 | DW | 91 x 74.5 cm | Unknown |
| The George Washington University Museum and The Textile Museum | 1977.35.9 | DW | 97 x 76 cm | Unknown |
| The George Washington University Museum and The Textile Museum | 1977.35.10 | DW | 97 x 77 cm | Unknown |
| The Museum of Fine Arts, Houston | 20.011.399 | DW | 95 x 77.5 cm | Pinilla, Ocucaje, Ica valley |
| Particular collection, Bolivia | No data | BW | No data | Isla del Sol Titicaca, Bolivia |
| Particular collection, Buenos Aires | 3 (Laboratory) | BW | 66.5 x 56 cm | Nevado de Chuscha. Salta, Argentina |
| University of Pennsylvania Museum of Archaeology and Anthropology | 27569 | TW | 80 x 71 cm | Pachacamac |
| Register of *Unku* located in museums in Europe, the United States of America and South America.  ^a^Black and White (BW), Inka Key (IK), Tocapu Waistband (TW), Diamond Waistband (DW), All Over Tocapu (AOT), Zigzag Waistband (ZZW). | | | | |
