# Supplementary material for: Inka Unku: Imperial or provincial? State-local relations
Source: PLoS One. 2023 Feb 8;18(2):e0280511. doi: 10.1371/journal.pone.0280511 (PMC9907846; doi:10.1371/journal.pone.0280511)
Supplement: S2 Table — Description of the 9 analytical parameters and 26 attributes. (DOCX) [file pone.0280511.s002.docx]

S2 Table. Standard analytical parameters and attributes. Description of the 9 analytical parameters and 26 attributes.

The 9 analytical parameters and its 26 attributes are arranged in numerical and alphabetical order.

| **Parameters 1. Material Selection** |
| --- |
| This parameter refers to the identification of fiber type (i.e., cotton and/or camelid-hair [llama, alpaca, guanaco, or vicuña]) and is characterized by a single attribute. |
| **Attribute 1a. Fiber type**. This can be identified macroscopically and/or microscopically. The most appropriate technique is to use scanning electron microscopy (SEM). Both fiber types (cotton and camelid) differ by their physical characteristics. Under low magnification (60x-120x), cotton is composed of individual flat, twisted and spiraling filaments in an assortment of S-and Z-turns. Camelid-hair, by contrast, is smooth, round and has no natural twists [1]. To identify the fibers microscopically, we applied the physical parameters suggested by Zoccola [2; i.e., length, fineness and color]. |
| **Parameters 2. Spinning** |
| Warp and weft yarns used in different areas of the textile. This parameter consists of 5 attributes. |
| **Attribute 2a. Yarn type***.* There are two large groups:  (a) simple: formed by one-color (monochrome) without decorative effects (regular).  (b) complex: formed by color variations (*mouliné* and *jaspé*), and twisting or different fiber inclusions (seeded [pre-columbian type], mélange, *flammée*, chenille, spiral, nub, crepe, frisé); [3]. |
| **Attribute 2b. Yarn length***.* To determine the length of yarn required for warp and weft in a quadrangle (W x H) based on its density.  D: Density in 1 cm^2^ (warp yarns/cm and weft passes/cm)  Warp  VTD: Vertical Thread Density/cm  X: number of warp threads  W: quadrangle width (cm)  H: quadrangle height (cm)  X= VTD x W  N_1_: warp yarn length (cm)= X x H    WEFT  HPD: Horizontal Passes Density/cm  Z: number of weft passes  W: quadrangle width (cm)  H: quadrangle height (cm)  Z= HPD x H  N_2_: weft yarn length (cm) = Z x W    This formula is replicated depending on the number of quadrangles the *unku* has and can be applied to each geometric section, the sum of these defining the total amount of yarn required to weave the entire piece. |
| **Attribute 2c. Yarn thickness or count**. Calculated by dividing the total length (X x Z) of the yarn (attribute 2b) by the weight (gr) of the *unku*. The higher the yarn count, the finer the yarn. For pre-Columbian yarns, Liliana Ulloa introduced a yarn-count range table, which is still used today by the Textile Laboratory of the Museo de la Universidad de Tarapacá, San Miguel de Azapa, Arica. The values range between very fine (35-30); fine (30-18); regular (18-10); thick (10-5); and very thick (5-0) [4-7]. |
| **Attribute 2d. Yarn structure***.* Layer sequence or yarn-structure order [1, 8] arranged as follows: first-order thread consisting of a single untwisted strand (S or Z); second-order thread consisting of two or more twisted strands (Z(2s) or S(2z)); third-order thread consisting of two or more second-order threads twisted and plied together S(2z(4s)) (or 4S-2Z-S); and fourth-order thread consists of two or more third-order plied threads and plied again Z(2s(4z(8s))) (or 8S-4Z-2S-Z). |
| **Attribute 2e. Degree of twist** This measures the angle of fiber inclination as a result of plying and is defined numerically between 0°, meaning no twisting, and 90°, meaning very twisted. The established ranges for measuring pre-Columbian fibers are as follows: loose: 0°-15°; medium: 15°-30°; strong: 30°-45°; very strong: over 45° [3, 6, 9-12]. |
| **Parameters 3. Color Selection** |
| Color selection parameters were organized using a weaving instrument consisting of a support (such as cane rods), wrapped in colored threads to create a color palette that would be used at the time of warping [13]. Aymara weavers refer to this today as *musa waraña* (combining colors [14]. This parameter consists of 2 attributes. |
| **Attribute 3a. Color name***.* Generic name assigned to each warp and weft yarn, preferably coded using the Munsell system, which defines three codes for color: hue, value, and chroma. |
| **Attribute 3b. Color Source**. Identification of natural or dyed colors. Raman spectroscopy, Fourier-transform infrared spectroscopy (FTIR), and gas chromatography/mass spectrometry are ideally used to analyze textile fiber dyes. |
| **Parameter 4. Loom Selection** |
| Selecting the type of loom depended on the size of the piece to be woven and the position adopted by the weaver in front of the textile. For the *unku*, a horizontal or vertical loom was used, allowing the piece to be viewed fully stretched out. This parameter consists of 2 attributes. |
| **Attribute 4a. Horizontal loom***.* Formed by four stakes nailed to the ground, separated according to the length and width of the textile. |
| **Attribute 4b. Vertical loom***.* Vertical wooden frame designed to secure the opposite ends of each warp to control tension. These looms could be used to weave pieces of up to 200 cm in width, necessary for certain Inka textiles and may have required two weavers working alongside one another simultaneously. |
| **Parameter 5. Preparation and Set Up** |
| Preparing and setting up the piece, which appears stretched out from the position of the weaver. This parameter consists of 4 attributes. |
| **Attribute 5a. Geometric shape***.* In the case of the *unku*, geometric shapes are limited to the following: trapezoidal, quadrangular, rectangular along the width, and rectangular along the length. |
| **Attribute 5b. Size***.* Inka tunics ranged from between 90-95 cm long to 75-77 cm wide [15-17]. |
| **Attribute 5c. Symmetry***.* Refers to whether the textile is symmetrical or asymmetrical. Total or mirror symmetry (horizontal and vertical) means that elements such as shapes, designs and colors are the same on both sides of the piece. Asymmetry indicates differences in shapes, designs or colors both horizontally and vertically of the textile [4, 6]. |
| **Attribute 5d. Spatial layout.** The piece is observed and register analytically ("read") fully stretched from the weaver's point of view, from one end to the center, including the following analytical sections: selvage, woven space where the piece begins and ends, generally distinguishable from the rest by a change of yarn type, diameter, or color. It is sometimes concealed by a particular finish; flange, which is the area located beside the selvage, and is larger in size and may be striped in some manner; *pampa* or flat space between the flange and center of the piece, often a single color; center, “heart” or *taypi,* as Aymara women call it, is the central space that divides or joins a piece together. The reverse face of the piece should be analyzed in the same way [18-20] (Fig 5). |
| **Parameter 6. Technological-Structural Construction** |
| A set of techniques to add structure to the weave, can vary densities and create designs according to the intentions of the weaver. This parameter consists of 2 attributes. |
| **Attribute 6a. Weaving structure**. Interlocking tapestry was the most widely used technique for the Inka *unku*, although with several variants, including eccentric wefts which served to make curvilinear designs, outline shapes, or break with geometric parameters and create curvature effects in color areas; additionally, openings were made using discontinuous warps [9:83, 15, 16, 21, 22]. |
| **Attribute 6b. Weave density***.* Number of warp threads and weft passes per cm^2^. This should be recorded at each change in color and weave structure. |
| **Parameter 7. Design and Decorative Effects** |
| Designs are produced by the arrangement of visible yarns and techniques applied to the surface of the textile. Effects are produced by applying yarn, twist, and density variations, or by varying the weave structure to produce effects or textures without altering the colors. The designs defined for the Inka *unku* (e.g., stripe compositions, checkerboard patterns, *tocapu*, diamonds and zigzags) are included as descriptive elements of this parameter, which comprises 6 attributes. |
| **Attribute 7a. Diamonds or rhomboids***.* Geometric shapes delimiting spatial zones with bilateral and/or quadripartite divisions. |
| **Attribute 7b. Stripes or bands.** Formed by variations in thicknesses, arrangement of contrasting colors or different shades. They may be based on pattern repeats, or have no discernible scheme, irrespective of whether natural, dyed, or mixed colors are used. |
| **Attribute 7c. Checkerboard**. Produced by alternating squares of contrasting colors, for example, a checkerboard pattern. |
| **Attribute 7d. *Tocapu****.* Design units, quadrangular and modular, arranged in multiple directions or in a different order within the textile, and enclosing a series of geometric or figurative motifs in a variety of colors and combinations. |
| **Attribute 7e. Zigzag***.* Continuous narrow band with half-diamond designs along the bottom edge of the tunics. Some *tocapu* display this same strip along the upper and lower margins. |
| **Attribute 7f***.* **Textures or effects**. Intentional elements created on the surface of the textile, such as bulging in color areas, variations in yarn type, changes in yarn density or twists, adding visual interest and haptic effects to the piece. |
| **Parameter 8. Finishes** |
| Techniques used to join parts of the textile and strengthen or determine their edges [23]. Types of finish are the only attribute for this parameter. |
| **Attribute 8a. Types of finish***.* The figure-eight stitch and zigzag-reinforced stitch that combines the displacement and density of the fishbone stitch. These attributes are mainly applied at the lateral seams. To reinforce selvages, overcast edges were used in different densities, and zigzag stitching was applied to the bottom. All these finishes appear mainly in *unku* from the Central Andes. Conversely, in valleys and along the Arica coast, the fishbone, overstitch that can be done in different densities and figure-eight stitches (in zigzag) were used for the neck or arm slits [6]. In both regions, we see examples of heading cords that lend structure and support to the piece, producing the same effect as a reinforced selvage. |
| **Parameter 9. Usages of the *unku*** |
| Conditions of the textile acquired throughout the three phases of its life, from its initial use to its reuse and disposal (e.g., in refuse dumps, tombs and offering pits). This analysis is based on the piece’s state of preservation from the moment it is excavated. Post depositional and taphonomic factors (e.g., physical, chemical, or biological transformations etc.) are also considered. This parameter consists of 3 attributes. |
| **Attribute 9a. Initial use***.* Original use, practical (e.g., for rulers, military personnel, bureaucrats) or ritual (e.g., as offering without being worn), as intended from the moment it was made. |
| **Attribute 9b. Reuse***.* Corresponds to the phase at which a piece left the hands of its original recipients to be reused for its useful life to be extended. This becomes more evident in features such as: holes, tears, stains, gaps, cuts, frayed edges, and folds. Some of these repairs may have entailed adding pieces of other textiles or reinserting or mending broken warp or weft threads. This analysis requires specifying the stitch type used for each repair and its location on the textile. |
| **Attribute 9c. Disposal***.* Refers to the moment a piece ceased to be worn or used, either to be deposited as an offering or garment in a tomb, or disposed of in a refuse dump, perhaps as it no longer fulfilled the function for which it was originally created. This includes fragments or complete pieces in different states of preservation and pieces used as unique ritual offerings (in tombs, pits, etc.) not in keeping with the funerary parameters of the site at which it was found. Consequently, at this stage, pieces may have been intentionally torn, burned, cut, or subjected to other alterations before disposal. |

**References**

1. Splitstoser JC. Weaving the Structure of the Cosmos: Cloth, Agency, and Worldview at Cerrillos, an Early Paracas Site in the Ica Valley, Peru. Washington, DC,: Department of Anthropology, The Catholic University of America; 2009.

2. Zoccola M. Fibras proteicas, pelos finos (camélidos y cabras): Identificación, caracterización, clasificación y condicionamiento. Cuaderno Tecnológico. 2014;14:1–44.

3. Agüero C. Tradiciones textiles de Atacama y Tarapacá presentes en Quillagua durante el periodo Intermedio Tardío. Boletín del Comité Nacional de Conservación Textil. 1998;3:103-28.

4. Correa J. Diseño de tejidos inspirados en piezas prehispánicas, que sirvan de patrones básicos para la creación de prendas textiles contemporánea [Proyecto título para optar al título de diseñador]. Valparaiso: Universidad de Valparaiso;1993.

5. Correa J, Ulloa L. Bolsas de la costa sur de Arica, periodo Tardío. Boletín de la Sociedad Chilena de Arqueología. 2000;29:9-19.

6. Carmona G. Caracterización de las prendas textiles incas presentes en sitios arqueológicos tardíos del extremo norte de Chile. [Memoria para optar al titulo de arqueóloga]. Santiago: Universidad de Chile; 2006.

7. Cases B. Bolsas de Quillagua: una sistematización del universo textil contenedor. Actas del XIV Congreso Nacional de Arqueología Chilena. Contribución Arqueológica 2. 1997, Copiapó: Museo Regional de Atacama; 2000. p. 83-118.

8. Splitstoser JC, editor The parenthetical notation method for recording yarn structure. Proceedings of the 13th Biennial Symposium of the Textile Society of America, Textile & Politics, September 19-22; 2012; Washington D.C.: Textile Society of America.

9. Emery I. The Primary Structures of Fabrics. London: Thames & Hudson Ltd; 2009[1966].

10. Agüero C. Clasificación de turbantes del periodo Formativo temprano. Boletín del Comité Nacional de Conservación Textil. 1994;2:61-70.

11. Cases B. Etnoarqueología y tecnología de las bolsas a la producción textil prehispánica. Un caso de estudio en Huancarane (período Intermedio Tardío, valle de Camarones) [Tesis para optar al grado de doctor en antropología]. Departamento de Antropología, Universidad de Tarapaca, Instituto de Investigaciones Arqueológicas Universidad Católica del Norte; Arica, San Pedro de Atacama; 2017.

12. Correa J. Descripción y análisis de diseño de los tejidos del Cementerio Oriente del valle de Quillagua. Boletín del Comité Nacional de Conservación Textil. 1998;3:129-44.

13. Arnold D, Espejo E. Andean weaving instruments for textile planning: The waraña coloured thread-wrapped rods and their pendant cords Indiana. Ibero-Amerikanisches Institut. 2012;29:173-200.

14. Arnold D, Espejo E. El Textil Tridimensional: la Naturaleza del Tejido Como Objeto y Como Sujeto. La Paz: Fundación Albó, Fundación Interamericana e Insituto de Lengua y Cultura Aymara (ILCA); 2013.

15. Jiménez Díaz MJ. Una “reliquia" inca de los inicios de la Colonia: El uncu del Museo de América de Madrid. Anales del Museo de América. 2002;10: 9-42.

16. Rowe AP. Technical features of Inca tapestry tunics. The Textile Museum Journal. 1978;17:5-28.

17. Martens T, Correa-Lau J, Santoro CM, Carter C, Cameron J. An Inka Unku from Caleta Vitor Bay, Northern Chile. Latin American Antiquity. 2021;32(1):201-8. Epub 2021/03/23. doi: 10.1017/laq.2020.81.

18. Gavilán V, Ulloa L. Proposiciones metodológicas para el estudio de los tejidos andinos. Revista Andina. 1992;10(1):107-34.

19. Zorn E. Un análisis de los tejidos en los atados rituales de los pastores. Revista Andina. 1987;52(2):489-525.

20. Cereceda V. Semiología de los textiles andinos: las talegas de Isluga. Chungara, Revista de Antropología Chilena. 2010;42(1):181-98.

21. Agüero C. Intervention in textile-making as a Tahuantinsuyu strategy for linking Northwest Argentina with the Atacama Desert (A.D. 1350-1500). Quaternary International. 2019;533:78-87.

22. O'Neale L, Kroeber AL. Textile periods of ancient Peru: I. Berkeley: University of California Publications in American Archaeology and Ethnology, Vol. 28; 1930.

23. Hoces de la Guardia S, Rojas AM. Vestimenta de mujeres en la nobleza Inca. Ajuar textil en el enterratorio del Cerro Esmeralda y sus relaciones con los textiles en miniatura de estatuillas. Nuevo Mundo - Mundos Nuevos. 2016;1(1). doi: DOI : https://doi.org/10.4000/nuevomundo.69833.
