# Supplementary material for: Inka Unku: Imperial or provincial? State-local relations
Source: PLoS One. 2023 Feb 8;18(2):e0280511. doi: 10.1371/journal.pone.0280511 (PMC9907846; doi:10.1371/journal.pone.0280511)
Supplement: S1 File — Supplementary description that characterize the CV unku. (DOCX) [file pone.0280511.s003.docx]

S1. File. CV *unku* patterns and attributes. Detailed supplementary description of the standard analytical patterns and attributes that characterized the Caleta Vitor Inka *unku*.

**Analytical pattern 1: Fiber-type identification**: optical and scanning electron microscopy (SEM-EDX) was used to observe yarn samples of warps, wefts and repairs from the *unku*. The samples were analyzed under low vacuum (Pa) and variable pressure (VP) mode at 150 Pa and in the column at a pressure of 2x10-5 Torr (high vacuum). The working distance used (WD) varied depending on the sample type. We used an acceleration voltage of 20 KV with a degree-of-twist range of 0° to 90°, while the images were taken at a resolution of 3024 x 2304 pixels at a scanning speed of 12 minutes and 54 seconds. To characterize the specimens, images were captured using SEM-EDX at magnifications of 800X, 1500X, 2500X; this allowed us to analyze the surface structure of the fibers, providing information on their morphology, surface, thickness, and pigmentation (Fig 1). To identify the camelid type and yarn color origin for the warps, wefts, and repairs, we compared the samples with yarns and fleeces from modern-day vicuñas and alpacas by observing the surface of the archaeological fibers.

|  |  |  |
| --- | --- | --- |
| a. Warp 1 and warp 2: beige - 800X magnification. | b. Weft 1, weft 4, and R1: dark brown - 1500X magnification. | c. Weft 3, weft 8, and weft 10: very dark brown - 2500X magnification. |
|  |  |  |
| d. Weft 2 and weft 7: white - 2500X magnification. | e. Weft 6: dark garnet red - 2500X magnification. | f. Weft 5: light garnet red -  2500X magnification. |
|  |  |  |
| g. Weft 9: medium brown - 2500X magnification. | h. R2: melange, very dark brown - 2500X magnification. | i. Modern-day natural vicuña  fleece - 1500X magnification. |
|  |  |  |
| j. Modern-day natural alpaca fleece - 1500X magnification. | k. Natural-dyed alpaca yarn with iron sulphate tooth - 1500X magnification. | l. Natural-dyed alpaca yarn without mordant - 1500X magnification. |

Fig 1. Fiber-Type identification through optical and scanning electron microscopy (SEM-EDX). (a-h) archaeological fibers of the CV *unku*; (i-l) modern-day fibers

In the image (a) shows a behavior consistent with that of cotton, i.e., short fibers in disarray and oriented in different directions. Images (b-h) show the fibers to be long, even, and orderly; they have a regular morphology, and the cuticles are thin, close together and overlapping, forming a wavy mosaic with a continuous pattern characteristic of camelid-hair fiber. The fibers in images (b, c, e, g and h) have a surface covering, probably the result of physical alterations by microorganisms (e.g., fungi), sediments, salts, or other extrinsic factors, or perhaps due to the surface adhesion of pigment remains. This hindered our capacity to clearly visualize the morphology and cuticles. Nevertheless, these irregularities show the sort of surface flakiness that can occur when colors are obtained from dyes. This includes the sample in image (f), although this has a smooth surface, and the regularity of its cuticle is visible. Clearly, white sample in image (d) differs from the others with its defined, regular, and evenly spaced cuticles. This is characteristic of the natural colors of alpaca fiber. In relation to the modern-day, non-archaeological samples, no significant differences can be observed between the morphological and surface characteristics of the naturally dyed sample with mordant in image (k) and that without in image (l). This allows us to affirm that the presence or non-presence of mordant in the fibers would not affect their surface qualities. Therefore, for the archaeological fibers, it would be very difficult to determine the presence of mordant using microscopic analysis alone.

In terms of fiber length, this could not be measured on an already woven piece. When measuring fiber thickness to determine quality, we were able to see that the fibers in images (c-e) had diameters of under 29 μm, falling within acceptable ranges for fine fibers. Fiber in image (f) has a thickness of 40.55 μm. Its relative thickness and light garnet-red color suggest it to be of poor quality.

For **analytical patterns 2 and 3: Spinning and color selection**, we were able to distinguish between warps that consisted of two different types of cotton-fiber yarn. The heading-cord yarns located at the top, bottom and center of the piece is monochrome/regular, of regular yarn count (10-18), 2^nd^ order thread with Z(2s) structure and strong degree of twist (30-45°); it has a natural beige color and a density of 2 yarns spanning 3mm. The structural warp yarn is monochrome, of regular yarn count (10-18), 2^nd^ order with Z(2s) structure and strong degree of twist (30-45°); it has a natural beige color and a density of 26 yarns per cm.

The alpaca-fiber weft yarns are located at the warp selvages, flanges (checkered), pampas and striped center. The yarn types are monochrome/regular, with a very fine yarn count (30-35), 2nd order S(2z) structure, and strong degree of twist (30-45°). The warp selvages have a density of 8 wefts over 2 warp yarns x 3mm and a natural, dark brown color. The black checkers are very dark/natural brown with a density of 48 wefts over 6 warp yarns x 1cm; the white checkers have a white/natural color with a density of 40 wefts over 6 threads x 1cm. The pampas consist of two colors: light/dyed garnet red with a density of 44 wefts over 6 warp yarns x 1cm, and dark/dyed garnet red with a density of 68 wefts over 6 warp yarns x 1cm. The striped pattern repeats consist of four colors: white/natural with a density of 32 wefts over 2 warp yarns x 7 mm; very dark/natural brown with a density of 28 wefts over 2 warp yarns x 7mm; medium/natural brown with a density of 28 wefts over 2 warp yarns x 7 mm and very dark/natural brown with a density of 28 wefts over 2 warp yarns x 7mm. This last yarn (very dark/natural brown) is repeated, forming the 4th color in the repeat pattern.

**Analytical patterns 4 and 5: Loom selection, set-up, and preparation of the piece.** Measuring over 80 cm in width, the *unku* was crafted using a vertical loom. From the wearer's viewpoint, this *unku* was conceived as a single piece, woven simultaneously in two equal and symmetrical parts (sides), with no distinction between the front and back. Furthermore, its two identical sides mean the piece is reversible. When worn, it is long and narrow: 91 cm in length from the base of the shoulders to about knee height for a person who is 1.6 m tall. In profile, the piece would reach the middle of the arm, measuring 41 cm from the base of the neck to elbow height. It has no sleeves. The spatial layout is described from the weaver's viewpoint and consists of structural ends (heading-cord yarns at the top and bottom selvages); checkered flanges; *pampa* formed by a plain (single color) area interrupted by a central (striped) diamond that is truncated at the corners.

**Analytical pattern 6: Technological-structural construction**. Consisting of the following weave structure: two shots of heading-cord yarns [1] at the top/bottom selvages and the discontinuous wefts at the neck slit in the middle of the piece (as viewed by the weaver), lending structure, weight, rigidity, and support. The interlocking tapestry weave [2] is the structural pattern that produces the checkerboard design and striped diamond which are worked into the plain section of the piece*.* Discontinuous warps are used to form the neck slit (26 cm). The intersection between the plain garnet-red area and the striped diamond shape is created using a slit tapestry weave [2:79, Fig 93]. Each weft pass links to the warp, clearly defining the edges of the different shapes. The textile is simultaneously woven in two equal parts, joined at the middle with grouped wefts using a 3/3 dovetail interlocking tapestry technique [2:80, Fig 97]. Where both pieces converge, there is a slight mismatch that is apparent from the difference in the alternating colors of the stripes that make up the diamond shape and pattern repeat.

**Analytical pattern 7: Design and decorative effects**. Produced by the checkerboard pattern, plain *pampa* with curvilinear effects/ eccentric weft and striped center. From the weaver's viewpoint, the checkers mark the start and end of the piece, arranged in 4 rows (columns when worn) of 9 squares each. The top and bottom of the piece begin and end with half squares, which, placed together, would form the column’s tenth square. The checkerboard pattern is created using two alternating colors (white and very dark brown); the white squares measure 9.0-10.0 cm wide x 7.5-9.5 cm high; the very dark brown squares are 8.5-9.0 cm wide and 7.5-9.3 cm high. Two of the squares on side B (2^nd^ and 4^th^ column) are one half very dark brown and the other half dark brown. Moving toward the center from the black-and-white checkers, there is a plain, garnet-red (dyed) area, which is interrupted by a rhomboid located in the center of the textile. The rhomboid is formed by a pattern repeat of plain bands (stripes when worn) with a regular width of between 4.0 -7.0 mm. The bands are white, very dark brown, medium brown, and very dark brown. To explain more clearly the pattern repeats, we have divided the diamond visually into 4 sections from the weaver's viewpoint. Lower section of side A to the neck slit: pattern repeats 12 times with 48 bands in total. Upper section of side B to the neck slit: pattern repeats 14 times and finishes in two colors (very dark brown/dark brown) with 59 bands in total. Lower section of side B to the neck slit: pattern repeats 11 times and finishes in two colors (white/very dark brown), with 46 bands in total. Upper section of side B to the neck opening: pattern is repeated 11 times and ends in three colors (white/very dark brown/dark brown) with 47 bands in total.

From the weaver's point of view, and once the piece has been folded in half to be worn, a half rhomboid is formed, simulating a breast plate that accentuates the neck. The half diamond or central “V” is truncated, leaving a margin of 4 cm out to the edges. Each warp selvage is formed or delineated by a thin (3 mm) dark brown stripe (from the wearer’s perspective, see Fig 5 in the main text). In *the pampa* (garnet red), a textured effect is produced by using the eccentric-weft technique to give a curvilinear, ribbed appearance (lower section, side A, from the weaver’s viewpoint). A further effect is created by varying the weft density to form broad bands, which are accentuated by tonal shifts in the garnet-red area with diagonal or “lazy lines” (upper section, side A, from the weaver's viewpoint). We also find the ribbing effect achieved by adjusting or tightening the wefts, the warps to stand out, forming a ribbed effect, because of their thicker diameter as a result of their three-ply structure. This effect is found in both garnet-red areas (*pampas*) on both sides A and B. We also observed loose, or “floating,” warps in various sections of the plain garnet-red area. This irregularity is found repeatedly and on both sides of the piece, with floats occurring regularly in every-other warp.

**Analytical pattern 8: Finishes.** Finishes were found at the neck-slit area and other areas on side A of the open tunic. Given it is an area subject to constant tension and pulling, the neck slit needs to be reinforced throughout the selvage. This is achieved using a simple blanket stitch. On the side B, there are two, white, camelid-fiber, yarn fragments (fine, S(2z), regular), with a double pass that would appear to be an overstitch, the rest of the lateral sides remain unjoined. This seems to have been for conservation purposes and the result of postdepositional actions.

**Analytical pattern 9: Usages** **of the *unku***. There are two repairs in the garnet-red *pampa* area (upper section, side B). We assume that the piece’s life history went beyond its initial use and that it was not disposed of at this stage. On the contrary, it was repaired to extend its useful life before being buried, at which time the piece began a new stage in its life history. The repairs are made from different yarns and colors. They do not overlap. Both are made using camelid-hair yarn and occur in the direction of the weft in the plain garnet-red area. One of these repairs was made with a medium, natural-brown, monochrome yarn (fine yarn count, in 2^nd^ order S(2z), with a strong degree of twist). The other is a mélange (mixed color), natural, very-dark-brown yarn with white fibers (fine yarn count, 2^nd^ order, S(2z), with a strong degree of twist). The repairs were made using a needle to weave the yarns into each other, imitating the original weave pattern so they became a structural part of the textile. Other signs of the *unku'*s life history are the warp losses and broken wefts, heavily marked folds, surface dirt and grease stains (side B, checkered area, and plain center). To this, we can add post-depositional marks such as wrinkles and the adhesion of dust, sand, and feathers, combined with a strong odor of decomposing marine products. We can also observe crosswise tears, possibly caused by having been unearthed during looting.

**References**

1. Hoces de La Guardia MS, Brugnoli P. Manual de Técnicas Textiles Andinas: Terminaciones. Santiago: Consejo Nacional de la Cultura y las Artes y Museo Chileno de Arte Precolombino; 2006.

2. Emery I. The Primary Structures of Fabrics. London: Thames & Hudson Ltd; 2009[1966].
