# Supplementary figures and images for: Inka Unku: Imperial or provincial? State-local relations
Source: PLoS One. 2023 Feb 8;18(2):e0280511. doi: 10.1371/journal.pone.0280511 (PMC9907846; doi:10.1371/journal.pone.0280511)

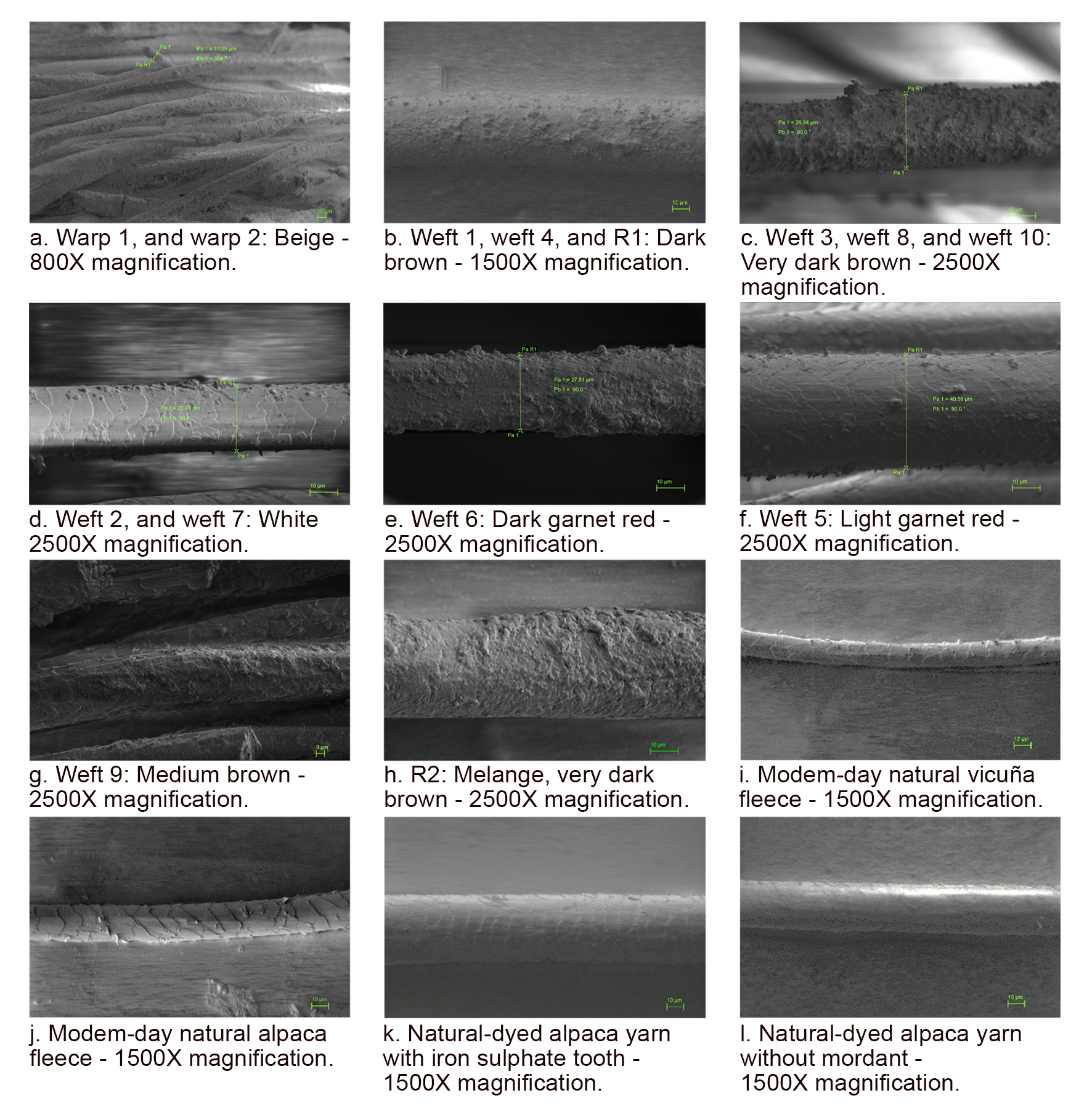

Supplement: S1 Fig — (TIF) [file pone.0280511.s004.tif]
