# Supplementary material for: Inka Unku: Imperial or provincial? State-local relations
Source: PLoS One. 2023 Feb 8;18(2):e0280511. doi: 10.1371/journal.pone.0280511 (PMC9907846; doi:10.1371/journal.pone.0280511)
Supplement: Attachment — Submitted filename: Response to reviewers.docx [file pone.0280511.s006.docx]

**PONE-D-22-00867/R1**: Inka Unku: Imperial or Provincial? State-Local Relations.

Review Comments to the Author

**Reply to Reviewer #1**:

***Query 1****: I recommend minor revisions before this is completely ready for publication. It would be very worthwhile to do a full copyedit review as there are a few places that need to be tended to for grammar, punctuation, sentence structure, or something along those lines. See, for example, lines 91-97; 105-108; 156-158; line 167; line 182; lines 224-226; 228-230; 244-245; 289-290; 302-303 (here also some of the content re: yarn spin could be put in a footnote, doesn’t all seem necessary here); lines 568-570. These are parts where I caught some kind of error or oversight but a professional copyeditor would be most useful.*

**Reply:** Agree and fixed. The text was reviewed and corrected in its entirety by a native English speaker

***Query 2***: *General comment: suggest that words like “state” and “imperial” be lower case across the board. There are still some inconsistencies in capitalization of these here and there and it would be cleaner to just lower case these words throughout, even re: “Inka state.”*

**Reply**: Partially agreed and fixed. The Associated Press (AP) and the Chicago Manual of Style, indicate that the word "state" is capitalized when referring to a government agency. In all other cases they prefer lower case. Therefore, we have opted for "Inka State" (and Inka Empire) but "state" (lowercase) for all other cases. Besides, different articles (mostly Plos One) speak of Inka/ Inca State. We maintained the Inka term capitalized because is a proper name.

***Query 3****:* *Line 53—what is the “importance of the relationship btwn state and local knowledge” what is the “dialectical arena in which state ideologies could be negotiated”*

**Reply**: Fixed. A long sentence has been added to deepen the conceptual terms pointed out, which stands as follows (see lines 53-62): In this regard, we address the importance of the relationship between state and local knowledge, which brought agents into a dialectical arena in which state ideologies could be negotiated. The importance lies in the fact that, despite the scale of the social and military structure of the Inka State, its expansive, economic and ideological success depended to a large extent on the agreements reached, peacefully or belligerently, with the local communities of the provinces integrated into the empire. Among the dialectical scenarios was the textile industry, in whose production the technical and idiosyncratic canons of the state were negotiated with those of the communities themselves. In this context, the *unku*, as symbols of imperial power, are key elements for materially visualizing the dialectical relationship between the state and the local communities.

For the analysis of this dialectical arena, we use the concepts of *metis* and *techne* [1] to facilitate an understanding of the dialectical relationship between these agents.

***Query 4****: Line 79: suggest removing first clause, re: “we recognize style …tool of social action” and beginning sentence with “The State-produced textiles” b/c what ‘style’ encompasses is vague here and subsequent sentences elaborate on a variety of things besides style that become ‘tools of social action’…technical aspects, manufacture/process, etc..*

**Reply:** Agree and fixed. The paragraph was rewritten and stands as follows (see lines 86-90): The technological styles and symbolic structures of the textiles produced for the state constituted an active tool of social action [5] insofar as their production depended on the weavers of the local communities who had to follow the state model. In this active role of perpetuating state canons, local weavers had the insight to introduce subtle technical and stylistic changes throughout Tawantinsuyu.

***Query 5****: Lines 109-121--these do not seem necessary to the text and maybe muddle the direction the discussion is headed in. If anything, the quoted text (re: “technology, as embodied…”) might be preserved but moved to a different section, prob. somewhere in previous pages;*

**Reply:** Agree and fixed. Part of the text of this paragraph was moved to a previous page, which stands as follows (see lines 101-106): Technology links social productive activities and their interactions with material culture [8], which means that “technology, as embodied material practice, is a socially charged and materially grounded arena in which agents express and negotiate social relationships, establish and express value systems, and give meaning to the object world” [9:162]. Technology, then, is recognized as a meaningful social activity, driven through agency processes [10].

***Query 6****: Line 168…sentence “This would explain,..” is confusing, would delete;*

**Reply:** Agree and fixed. The full sentence was deleted.

***Query 7****: Line 196 “discontinuous warps and wefts …woven for the elite” is a confusing phrase;*

**Reply:** Agree and fixed. The sentence was rephrased as follows (lines 203-206): Discontinuous warps and wefts were presumably woven “to signal the office of the person who wore that class of garments” [48:230]. Consequently, Dransart, has suggested that “it is possible to see different levels of social articulation in these garments” [48:230].

***Query 8****: Line 469…the different ‘stages’? vs. ‘uses’ of a textile’s life history?;*

**Reply:** Agree and fixed. The sentence was rephrased as follows (lines 475-476): This analytical process makes it possible to identify the different stages of the textile's life history [101].

***Query 9****: Lines 546-550—this is so interesting about the half dark brown/half very dark brown squares, but can there be elaboration here as to why this would be local hallmark versus other explanation? Do we see examples of this kind of chromatic duality locally, that can be pointed to here for support? Also, the statement is that these half-squares are not conspicuous but they’re located or seem to be located toward the center of the textile panel, which would seem to be more conspicuous than not (?)*

**Reply:** We particularly appreciate this comment, and we, added the following phrase to clarify this issue (lines 554-566): This characteristic of two squares with chromatic duality in the brown range is exemplified on a large scale (i.e., the entire surface of the textile) in two *unku* defined as provincial in the collection of the Ethnological Museum of Berlin (VA 16630 and VA 4576) [105]. Another case, but with curved figures, is observed with a duality of blue range (VA 62696). The CV *unku*, a piece that integrates imperial and provincial morphologies, technologies, and styles, is a perfect example of syncretism, which transforms it into a local product. The detail of the chromatic duality in a single square of a checkered surface has not been noted in any other *unku* of the BW type, with which this piece would be associated. Therefore, we suggest investigating this feature as an element of the creation of local weavers who, following the state templates, introduced distinctive hallmarks. As for the prominence of this feature, although it is located on the front flange of the garment, due to slight tonal differences, it is not readable. Similarly, it occurs with the intersection of the stripes at shoulder height, which are in less conspicuous positions. We identified both features as having low-visibility.

***Query 10****: Line 566, a little unclear-- what is the ‘new’ role that the mending presumably highlights, apart from the original role the piece would have had before the mending…*

**Reply:** The whole paragraph was rewritten as follows (lines 577-587): Repairs to the piece were made by intricately and delicately emulating the original technique. As such, they are not immediately apparent and would have been barely perceptible to observers. For this careful repair work, the mender used composite yarns of different colors to those of the original textile, thus disclosing the intention to modify the conservation of the piece and unveil his hallmark as a mender. The repairs were intended to prolong the lifespan of the piece and thus give way to the second usage (reuse) of its life history. The CV *unku* was a singular piece in which, at first glance, state and provincial canons are identified, which thus made it an object valued locally and representative of a social, cultural, geographical, and temporal context. In this sense, this piece was transformed into an inheritable and desirable object given its ideological and political charge, which was to be maintained in perpetuity through reparations, thus endorsing the position of the character or family lineage in which the piece fell, to the extent that it continued to be part of the structure of the political relationship between the state and this local community.

***Query 11****:* Line 621—in the context of broader Andean cultural practices, another way of saying this might be the ‘tradition of giving and receiving ‘coopted’ by the state” rather than “‘established’ under the state”

**Reply:** Agree and fixed.

***Query 12****: Line 626-628 and ‘syncretic conditions’ in textiles that cannot be found or repeated in textiles—but in other parts of the paper the comment seems to be that ceramics can also display hallmarks of local tradition along with state aspects (e.g. pp. 11, 27)*

**Reply:** Agree and fixed. The sentence was rephrased as follows (lines 646-649): This syncretic condition of the textile could also be repeated in other goods produced by and for the state. For example, despite the rigid production processes of ceramics, artisans managed to introduce technical and aesthetic variations that resulted in a diversity of vessels defined as local or provincial Inka.

***Query 13****: Line 632—but what comes up in the content is that not all are ‘masked hallmarks’...that should be included here for clarity/nuance*

**Reply:** Agree and fixed. The sentence was rephrased as follows (lines 652-654): The tension embodied by weavers in having to explicitly reproduce state patterns, and mask or subtly unveil their local hallmarks are well represented and expressed in this *unku*.

**Reply to Reviewer #3:**

***Query 1****:* Thank you for your detailed and careful attention to the revisions. It is now a well-written, clearly organized, and tightly focused argument. I look forward to seeing the final product in print. Please note though the phrase "Error! Reference source not found" that appears in line 401 (page 18). That is the only one I found.

**Reply:** Agree and fixed.
